# What Makes Artificial Intelligence Exceptional in Health Technology Assessment?

**DOI:** 10.3389/frai.2021.736697

**Published:** 2021-11-02

**Authors:** Jean-Christophe Bélisle-Pipon, Vincent Couture, Marie-Christine Roy, Isabelle Ganache, Mireille Goetghebeur, I. Glenn Cohen

**Affiliations:** ^1^ Faculty of Health Sciences, Simon Fraser University, Burnaby, BC, Canada; ^2^ Faculty of Nursing, Laval University, Quebec, QC, Canada; ^3^ École de Santé Publique, Université de Montréal, Québec, QC, Canada; ^4^ Institut National D’Excellence en Santé et en Services Sociaux (INESSS), Montréal, Québec, QC, Canada; ^5^ Harvard Law School, Cambridge, MA, United States

**Keywords:** artificial intelligence, exceptionalism, ethical, social and legal implications, health technology assessment, health regulation

## Abstract

The application of artificial intelligence (AI) may revolutionize the healthcare system, leading to enhance efficiency by automatizing routine tasks and decreasing health-related costs, broadening access to healthcare delivery, targeting more precisely patient needs, and assisting clinicians in their decision-making. For these benefits to materialize, governments and health authorities must regulate AI, and conduct appropriate health technology assessment (HTA). Many authors have highlighted that AI health technologies (AIHT) challenge traditional evaluation and regulatory processes. To inform and support HTA organizations and regulators in adapting their processes to AIHTs, we conducted a systematic review of the literature on the challenges posed by AIHTs in HTA and health regulation. Our research question was: What makes artificial intelligence exceptional in HTA? The current body of literature appears to portray AIHTs as being exceptional to HTA. This exceptionalism is expressed along 5 dimensions: 1) AIHT’s distinctive features; 2) their systemic impacts on health care and the health sector; 3) the increased expectations towards AI in health; 4) the new ethical, social and legal challenges that arise from deploying AI in the health sector; and 5) the new evaluative constraints that AI poses to HTA. Thus, AIHTs are perceived as exceptional because of their technological characteristics *and* potential impacts on society at large. As AI implementation by governments and health organizations carries risks of generating new, and amplifying existing, challenges, there are strong arguments for taking into consideration the exceptional aspects of AIHTs, especially as their impacts on the healthcare system will be far greater than that of drugs and medical devices. As AIHTs begin to be increasingly introduced into the health care sector, there is a window of opportunity for HTA agencies and scholars to consider AIHTs’ exceptionalism and to work towards only deploying clinically, economically, socially acceptable AIHTs in the health care system.

## Introduction

Health technology assessment (HTA) is key to the introduction of artificial intelligence (AI) applications in health. HTA generally requires a systematic examination of health technologies’ features, effects, and/or impacts allows for the appraisal of clinical, economic, social, organizational and ethical implications (Banta and Jonsson 2009; Kristensen et al., 2017; O’Rourke et al., 2020). While regulatory assessment often is conducted by supranational (e.g., European Medicines Agency, EMA) and national (US FDA, Health Canada) regulators, HTA is mostly conducted at regional, provincial or state-based level and represents the main gateway for a health technology (e.g., drugs, vaccines, medical devices) to be widely administered to patients (Vreman et al., 2020; Wang et al., 2018). A health technology that is positively evaluated by a health regulator or an HTA agency signals significant support for its use, causing clinicians, patients, hospital administrators and third-party payers (such as public or private health insurers) to consider deploying and reimbursing this technology in their health care system or setting (Allen et al., 2017; Wild, Stricka, and Patera 2017). However, AI is not just another health technology, and many commentators view its assessment as complex and particularly challenging (Harwich and Laycock 2018; Mason et al., 2018; Shaw et al., 2019). For instance, AI health technologies (AIHT) implementation within the healthcare system is often done in a fairly short timeframe after their development (months rather than years as for drugs and vaccines), with the result that there is not yet as much evidence of their effectiveness and impacts as would be required by traditional HTA for many other health technologies (Babic et al., 2019). Moreover, AI systems deployed within the healthcare system continue to learn and evolve over time based on the data they process (Reddy 2018); this is in contrast, for example, with drugs whose formulation, dosage and routes of administration are regulated, and to be modified for use in clinical context and service delivery, often require new approval by HTA. In addition, AI systems require to be trained on and use vast amounts of (potentially sensitive) data (about patients, research participants, clinicians, managers, health care systems, etc.) that raise issues of privacy, (cyber)security, informed consent, data stewardship and control over data usages (Wang and Preininger 2019; Dash et al., 2019; Sun and Medaglia 2019; Bartoletti 2019).

The application of AI in health is expected to transform the way we diagnose, prevent and treat as well as the way we interact with technologies (Patel et al., 2009; Hamet and Tremblay 2017; The Lancet 2017). This may advance healthcare by enhancing efficiency by automatizing routine tasks and decreasing health-related costs (Shafqat et al., 2020), broadening access to healthcare delivery (Harwich and Laycock 2018), targeting more precisely patient needs (Jameson and Longo 2015), and assisting clinicians in their decision-making (Lysaght et al., 2019; Smith 2020). For these benefits to materialize, governments and health authorities must efficiently regulate AI, and conduct appropriate health technology assessment (HTA). However, the very definition of AI in health is still the subject of discussion, debate and negotiation among both researchers and government authorities. AI in the health sector can be broadly defined as a field concerned with the development of algorithms and systems seeking to reproduce human cognitive functions, such as learning and problem-solving (Tang et al., 2018) with (current and anticipated) uses that include (without being limited to) supporting medical decision-making (Ahmed et al., 2020), pharmacovigilance (Leyens et al., 2017), and prediction and diagnosis (Noorbakhsh-Sabet et al., 2019). In fact, some AIHTs have already been approved by the FDA, such as AI-powered devices to diagnose eye diseases (Samuel and Gemma Derrick 2020). Risks and harms of AI in healthcare are described at all levels, from the clinical encounter (e.g., adverse effects of an AIHT that can spread to entire patient populations, inexplicability of an AI-based medical decision, issues with assigning responsibility for adverse events, and patients’ loss of trust in their provider) to society as a whole (e.g., furthering inequalities due to algorithm training on biased data) (Sparrow and Hatherley 2019). Interestingly, one indication that current HTA processes are not yet well adapted is the fact that a significant number of AIHTs are benefiting from regulatory fast-track and do not undergo HTA review, a situation that is particularly noticeable in the United States (Benjamens et al., 2020; Gerke et al., 2020; Tadavarthi et al., 2020).

Even though AI solutions offer great potential for improving efficiency, health organizations are confronted with a vast array of AI solutions that have not yet been subject to extensive HTA (Love-Koh et al., 2018). Moreover, many authors have highlighted that these new technologies challenge traditional evaluation processes as well as the assessment of the ethical, legal and social implications (ELSI) that AIHTs may entail (He et al., 2019; Racine et al., 2019; Shaw et al., 2019; Ahmad et al., 2020; Benjamens et al., 2020), thus further impeding the already insufficient evaluative processes of AI health technologies (AIHTs). To inform and support HTA organizations in adapting their evaluation processes to AIHTs, we conducted a systematic review of the literature on the ethical, legal and social challenges posed by AIHTs in HTA. The present article was guided by this question: what makes artificial intelligence exceptional in health technology assessment? To our knowledge, this is the first review on this topic. After describing the methodology of the review, we will provide a comprehensive overview of AI-specific challenges that need to be considered to properly address AIHTs’ intrinsic and contextual peculiarities in the context of HTA. This will lead to point possible explanations of this exceptionalism and solutions for HTA. Overall, this review is intended to build insights and awareness and allow to inform HTA practices.

## Methodology

To map the exceptional challenges posed by AIHTs in HTA, we conducted a literature search for articles indexed in PubMed, Embase, Journals@Ovid, Web of Science and the International HTA database. Our review is part of a larger literature review addressing the full range of ethical, legal, social and policy implications that impact HTA processes for AIHTs. Therefore, the search strategy focused on three concepts: AI, HTA and ELSI. Table 1 presents the search equations by theme for each reviewed database. In terms of definition of AI, we sought to remain agnostic and did not use specific definitions of AI. Instead, we used an inductive approach using a series of keywords (see Table 1) to identify and collect articles that mention using or discussing AIHTs. The construction of the research strategy and the choice of equations was supported by librarians.

**TABLE 1:** Search strategy.

Concepts	Terms
**AI**	**PB** = [(Artificial Intelligence) OR (Machine Learning) OR (Deep Learning) OR (Natural Language Processing) OR (Chatbot*) OR (Carebot*) OR (Big Data)] OR [Artificial intelligence OR Big Data (MeSH Terms)]
	**EM; OJ; WoS** = (Artificial Intelligence) OR (Machine Learning) OR (Deep Learning) OR (Natural Language Processing) OR (Chatbot*) OR (Carebot*) OR (Big Data)
	**iHTAd** = (Artificial Intelligence) AND
**HTA**	**PB** = (Health Technology Assessment) OR (HTA) OR (Technology Assessment) OR [Technology Assessment, Biomedical (MeSH Terms)]
	**EM; OJ; WoS** = (Health Technology Assessment) OR (HTA) OR (Technology Assessment)
	**iHTAd** = [*Empty*] AND
**ELSI**	**PB** = (ESLI) OR (Ethic*) OR (Bioethic*) OR (Moral*) OR (Legal*) OR (Law) OR (Societ*) OR (Polic*) OR (Governance) OR (Trust) OR (Mistrust) OR (Jurisprudence) OR (Public Policy) OR (Bioethics OR Ethics OR Jurisprudence OR “Public Policy” [MeSH Terms])
	**EM; OJ; WoS** = (ESLI) OR (Ethic*) OR (Bioethic*) OR (Moral*) OR (Legal*) OR (Law) OR (Societ*) OR (Polic*) OR (Governance) OR (Trust) OR (Mistrust) OR (Jurisprudence) OR (Public Policy)
	**iHTAd** = [*Empty*]

**Legend**. PB = PubMed; EM = Embase; OJ = Journals@Ovid Full Text; Databasel; WoS = Web of Science; iHTAd = International HTA.

**FIGURE 1:**
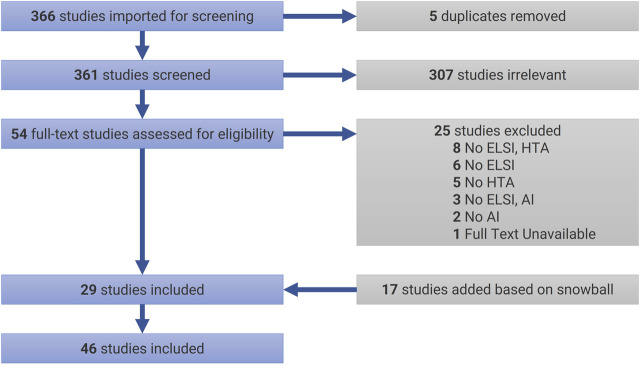
PRISMA Flowchart. AI = artificial intelligence; ELSI = ethical, legal, and social implications; HTA = health technology assessment.

The initial search (as of December 27, 2020) returned a total of 366 articles, which were uploaded in Covidence. JCBP and VC conducted a careful analysis of the titles and abstracts that lead to excluding 307 articles, and JCBP conducted the subsequent analysis of main texts allowed to select 29 articles for review (see Table 2 for selection criteria). In case of doubt or ambiguity, articles were discussed with MCR to decide on inclusion or exclusion. In addition to this sample, in January 2021, a snowball process helped identify 17 additional papers that fitted the selection criteria. Figure 1 presents our review flowchart following PRISMA’s guidelines (Moher et al., 2009). Documents were thematically analyzed (Braun and Clarke 2006) with the help of NVivo 12. In the present article, we focus on the theme “exceptionalism of AI in HTA”. Additional themes will be published in subsequent papers.

**TABLE 2:** Selection criteria.

	Specifics
Date	2016–2020 (5 years)
Language	English; French
Study design	Descriptive; Experimental; Opinion/Perspective; Empirical Research; Literature Review
Type of publication	Original research; Commentary; Editorial

## Results

**FIGURE 2:**
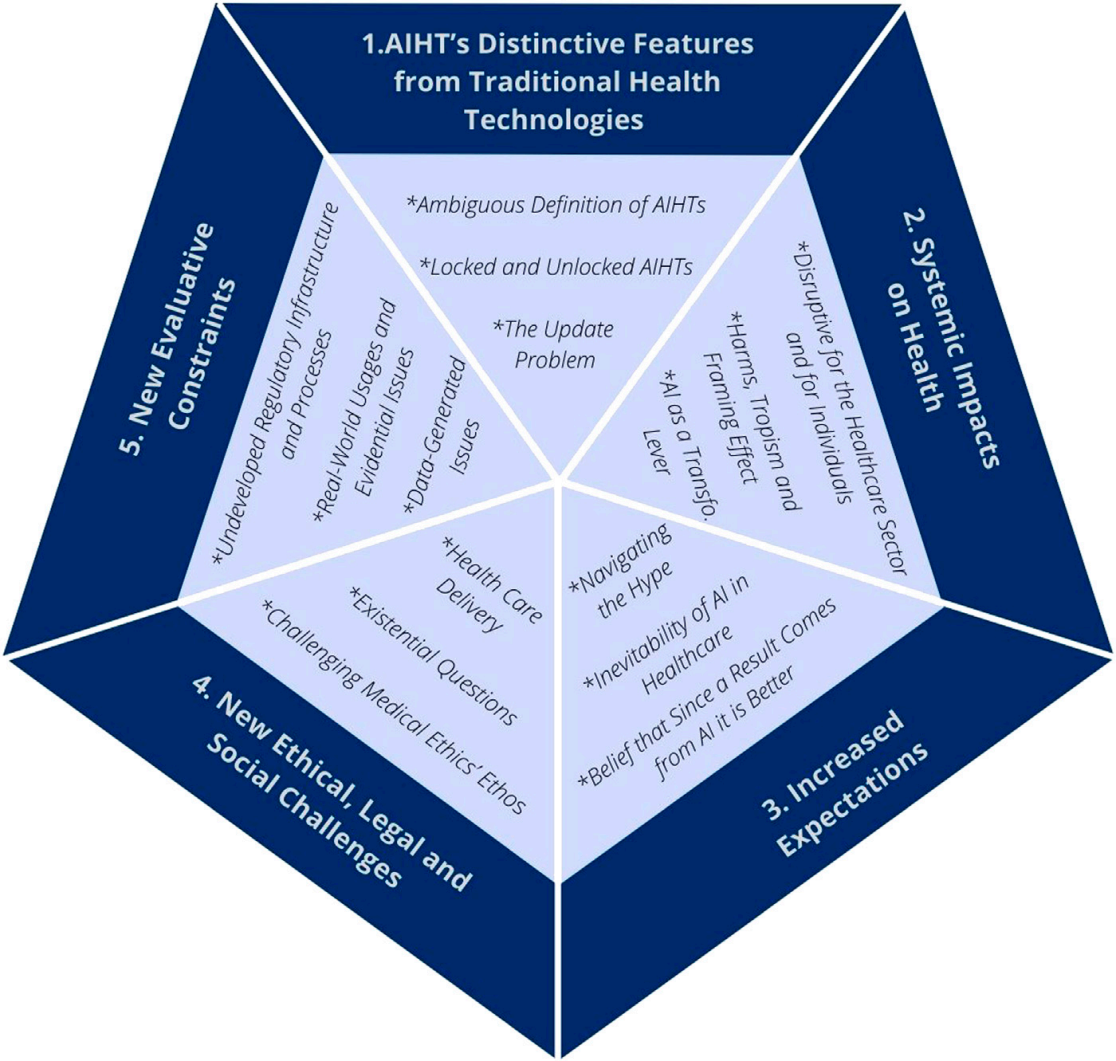
The five main aspects of artificial intelligence health technologies’ exceptionalism.

What follows is a presentation of the key considerations that have been raised in the reviewed literature regarding AI’s peculiarities, and the challenges they raise, in the context of HTA. Twenty eight articles from the total sample discussed these peculiarities and challenges, which are presented as exceptional features of AI by authors. The “exceptionalism” of AIHTs can be broken down into five main aspects (see Figure 2): 1) AIHT’s distinctive features; 2) their systemic impacts on health care and the health sector; 3) the increased expectations towards AI in health; 4) the new ethical, social and legal challenges that arise from deploying AI in the health sector; and 5) the new evaluative constraints that AI poses to HTA. Table 3 presents a summary of the key considerations for each aspect.

**TABLE 3:** A summary of the five main aspects of AIHT’s exceptionalism.

	Key considerations (In italic key sub-considerations)	Examples from the reviewed sample
1. AIHT’s Distinctive Features from Traditional Health Technologies	AIHTs are different from traditional health technologies because of their capacity to continuously learn, their potential for ubiquity throughout the health care system, the opaqueness of their recommendations and the ambiguity of their definition (*Ambiguous Definition of AIHTs*)	Locked AIHTs could become outdated potentially from the moment they are prevented from evolving. Thus, locking AIHT may cause it to become outdated and increase chance of contextual bias in real-life contexts
	Locked algorithms will always yield the same result when it is fed by the same data. They are not *per se* safer and may require new regulatory approvals, though they are easier to assess than unlocked algorithms. Unlocked or adaptive algorithms improve over time, which demands that their safety and security must be continually re-evaluated. ‘Lifecycle’ regulation seems to be key in addressing these concerns, but for the most part burden lies on the regulators to adjusted their assessment of an AIHT in light of the evolving evidence, which is very resource intensive and for which HTA agencies are not yet equipped to conduct. (*Locked and Unlocked AIHTs*)	
	Algorithms will need to be regularly updated (at high or even prohibitive prices) due to advances in medical knowledge and access to new datasets or at the risk of their usage becoming malpractice. Updating or replacing an AIHT will involve additional post-acquisition costs to the clinics and hospitals that purchased them. The difficulty of managing the consequences of an outdated algorithm outweighs those of a drug or other health product that must be withdrawn from the market (*The Update Problem*)	
2. Systemic Impacts on Health	AI may have systemic effects that can be felt across an entire health care system, or across health care systems in several jurisdictions, initiating extensive and lasting transformations that are likely to affect all actors working in, using or financing the health system. In addition, AIHTs can have systemic real-world consequences for patients and non-ill or non-frequent users of the health care system. However, AI will not address everything that has to do with the overall well-being of people (*Disruptive for Both the Healthcare Sector and for Individuals*)	AI’s role in health surveillance, care optimization, prevention, public health, and telemedicine will cause AIHTs to affect non-ill or non-frequent users of the health care systemAn AIHT trained on medico-administrative data in a context where physicians have often modified their billing to enter the highest paying codes for clinical procedures would cause the algorithm to infer that these codes represent the usual, standard, or common practice to be recommended, thus introducing a bias in the algorithm and leading to a cascade of non-cost effective recommendations
	Mistakes due to AIHTs used in clinical care and within the health care system have the potential to widely affect the patient population, suggesting that it is all the more necessary that all algorithms should submitted to extensive scrutiny. In addition, “tropic effects” (i.e., code embedded propensity towards certain behaviors or effects) may increase the risk of inappropriate treatment and care, and may result in importing AIHT-fueled standards and practices that are exogenous and non-idiosyncratic to local organizations. Furthermore, the large-scale systematization of certain behaviors may end up resulting in significant costs and harms (*Harms, Tropism and Framing Effect*)	
	Some authors suggest AIHTs should be regarded as a “health system transformation lever” for improving health care and a key enabler of learning healthcare systems (LHS) (*AI as a Transformation Lever for the Health Sector*)	
3. Increased Expectations	The “automation bias” describes the belief that an AI-generated outcome is inherently better than a human one. This is reinforced by the technological imperative, i.e., the pressure to use a new technology just because it exists (*Belief that Since a Result Comes from AI it is Better*)These high expectations toward AIHTs form the basis of the inevitability of AI in health. However, the concept of *AI chasm* refers to the phenomenon that while AIHTs are very promising, very few will actually be successful once implemented in clinical settings and can help rebalance the expectations. HTA agencies have an important role to play here to contain this phenomenon (*Inevitability of AI in Healthcare*)	The adoption and impact of AIHTs are unlikely to be uniform or to improve performance in all health care contexts because of the technology’s distinctive features, its systemic effects on health care organizations and the human biases associated with the use of these technologies. AIHTs can significantly affect and highlight particularities of workflow and design of individual hospital systems, causing them not to respond in an intended way. Therefore, AIHTs represent great challenges for deciding whether marketing authorization is justified
		
	AI is currently in an era of promises rather than of fulfillment of what is expected from it. Possible consequences of this hype can be very significant but HTA agencies and regulators have an important role to play (*Navigating the Hype*)	
4. New Ethical, Legal and Social Challenges	AIHTs present new ethical, legal and social challenges in the context of health care delivery; by calling into question the roles of patients, HCPs and decision-makers; and by conflicting with medicine’s ethos of transparency	Patients who compare very well with historic patient data will be the ones benefiting the most from AIHTs, calling for caution with regards to patient and disease heterogeneity
	Key AIHT-stemmed ethical challenges in care delivery are: AI-fostered potential bias; patient privacy protection; trust of clinicians and the general public towards machine-led medicine; new health inequalities (*Health Care Delivery*)	Practical and procedural ethical guidance for supporting HTA for AIHTs has not yet been thoroughly defined. For instance, distributive justice role in HTA for AIHT is not well specified
	AI being unlike most other health technologies, it forces the questioning of the very essence of humans. It also raises new existential questions regarding the role of regulators and public decision-makers AIHTs unparalleled autonomy intensifies ethical and regulatory challenges (*Existential Questions*)	AI-stemmed existential questionning includes the reflection that more and more clinicians are having about the proper role of healthcare professionals and what it means to be a doctor, a nurse, etc. And from the patients’ perspective, what it means to be cared for by machines and to feel more and more like a number in a vast system run by algorithms
	AIHTs are often opaque, which poses serious problems for their acceptance, regulation and implementation in the health care system. AI’s benefits for health care will come at the price of raising ethical issues specific to the technology (*Challenging Medical Ethics’ Ethos*)	
5. New Evaluative Constraints	AIHTs raise new evaluative constrains at the technological level due to the data and infrastructure required (*Data-Generated Issues*)New constraints also appear at the clinical level because of the greater variation in AIHTs performance between the test environment and the real-word context than those of drugs and medical devices (*Real-World Usages and Evidential Issues*)	The adoption and impact of AIHTs are unlikely to be uniform or to improve performance in all health care contexts because of the technology’s distinctive features, its systemic effects on health care organizations and the human biases associated with the use of these technologies. Therefore, AIHTs represent great challenges for deciding whether marketing authorization is justified, and it forces to question whether marketing authorization at the 10,000 foot level for the product is appropriate and efficient as opposed to for more specific uses closer to the impacted communities and the point of delivery
	This high level of complexity requires a special regulation of AIHT, specifically adapted to its complexity (*Undeveloped Regulatory Infrastructure and Processes*)	

### Artificial Intelligence Health Technologies’ Distinctive Features From Traditional Health Technologies

AIHT’s exceptionalism is associated with the technology’s definitional and foundational nature. Distinctive features include AIHTs’ ambiguous definition; the fact that AIHTs may or may not continue to evolve; and the need to keep AIHTs up to date to reap the benefits and avoid the risks of harms.

#### Ambiguous Definition of Artificial Intelligence Health Technologies

According to Gerke et al. (2020), AIHTs are different from traditional health technologies for three reasons: their capacity to continuously learn, their potential for ubiquity throughout the health care system, and the opaqueness of their recommendations. However, AIHTs suffer from ambiguities with respect to their definition and purpose as there is no agreed-upon definition that may help build an adapted and efficient policy and regulatory infrastructure (Pesapane et al., 2018). The drawback of AI exceptional features and of the high variability that exists among AI systems is that it poses definitional problems that affect AIHTs’ regulation and slow down their deployment in the healthcare sector (Love-Koh et al., 2018; Haverinen et al., 2019). Compared with traditional health technologies (such as drugs, vaccines and medical devices), AIHTs are not static products and have the capability to learn and improve over time (Parikh, Obermeyer, and Navathe 2019; Dzobo et al., 2020). AIHTs are therefore in stark contrast with most technologies in medicine, which are fairly well defined and usually implemented when they are fairly well understood.

#### Locked and Unlocked Artificial Intelligence Health Technologies

Contributing to the distinctiveness of AIHTs in the health sector, self-learning and self-adaptation propensities clash with current regulatory frameworks and clinical practices (Alami et al., 2020; Fenech et al., 2020). It is easier to evaluate “locked” AIHTs, which are much more comparable to current health technologies (which cannot by themselves evolve). Currently, the majority of FDA-approved AIHTs have their capability to evolve locked (Dzobo et al., 2020; Miller, 2020). A locked algorithm will always yield the same result when it is fed by the same data, therefore it does not change overtime with uses. Locked algorithms are not *per se* safer. They could be more harmful than “unlocked” or “adaptive” algorithms if they end up yielding erroneous results (based on legacy training data that are outdated), misleading patient care or systematizing biases (Prabhu 2019). Thus, a locked AIHT may require new regulatory approvals if during real-world usage significant and unexpected patterns of results are observed (i.e., stable and expected process produces outcomes unexpected because of incorrect priors about the data fed into an AIHT) or if it is deemed necessary to update the algorithms to match advances in medical knowledge (Gerke et al., 2020; Miller, 2020). There also is the issue of when AIHT is used (or not used) on new populations that differ from the training data, which raises questions about how the training data upon which an AIHT was developed and whether certain populations may be unduly excluded from benefiting from its development and implementation. Therefore the concept of locked may be misleading and should not be conveyed as safer (Babic et al., 2019). Unlocked or adaptive algorithms that improve over time is the future according to some (Prabhu 2019) as they will outperform humans (Dzobo et al., 2020). But some issues are to be expected. Unlocked AIHT may change as they process new data and yield new outcomes without the knowledge or oversight of its users, which demands that their safety and security must be continually re-evaluated (Abràmoff et al., 2020). Also, unlike traditional healthcare technologies and locked algorithms, unlocked AIHTs are more vulnerable to cyber-attacks and misuse that can cause the algorithm to generate problematic and highly damaging outputs (Babic et al., 2019; Miller, 2020).

#### The Update Problem

Another consideration that helps AIHT qualify for being an anomalous technology in the health sector is that the algorithms will need to be regularly updated (at high or even prohibitive prices) due to advances in medical knowledge and access to new datasets or at the risk of their usage becoming malpractice. To allow a rigorous analysis of the safety, efficiency, and equity of a given AIHT, it is necessary that the locked or unlocked state of the algorithm is always known to regulators and end-users (Char et al., 2020). Such transparency is necessary since due to the very distinct ethical and clinical implications that locked or unlocked AIHTs may generate. The update problem implies that a locked AIHT could quickly become outdated—potentially from the moment it is prevented from evolving (with or without supervision)—and that this could generate important risks as a result of the deployment and use of AIHTs in real-life contexts (Abràmoff et al., 2020). Although not all algorithms may need to evolve or be updated in the short term, at some point in time, updating or replacing an AIHT will involve additional post-acquisition costs. Post-acquisition updates and costs may seem counter-intuitive considering the distinctive characteristics attributed to AI, such as self-learning and continuous improvement. This may lead for certain organizations (in particular, in less affluent contexts or in periods of economic turmoil) not to deploy updates which will result in the uses of outdated algorithms and therefore sub-optimal benefits (if not harms) for some patients or services (Prabhu 2019). Since AIHT are considered as being more pervasive than physical technologies (such as drugs and other health products), some are arguying that managing the consequences of an outdated algorithm outweigh those of traditional health technologies (Babic et al., 2019; Prabhu 2019); even if it is very difficult to withdraw effectively a drug from the market, it is still possible to do so, while it may be much more challenging for AIHTs that are less visible, interpretable and tangible and more likely to be embedded in a hospital’s or health system’s IT systems.

### Systemic Impacts on Health

Characteristic of disruptive technologies, AIHTs are said to have significant and systemic impacts on the healthcare sector. From the outset, what emerges is that AI has a capacity for information analysis that surpasses what is currently available from health professionals, healthcare managers or even from learning health systems (LHS) (Cowie 2017; Pesapane et al., 2018; Char and Burgart 2020). AI is geared towards changing healthcare practices by facilitating a better integration of innovations and of best practices that will yield optimal care delivery (Grant et al., 2020). These systemwide impacts may lead to both risks of harms and opportunities to optimize the health care system that must be taken into consideration in HTA.

#### Disruptive for Both the Healthcare Sector and for Individuals

Contrary to many health technologies, AI may have systemic effects that can be felt across an entire health care system, or even more so across health care systems in several jurisdictions (Dzobo et al., 2020). Gerhards et al. (2020) go as far as stating that AIHTs (especially those using machine learning) can yield significant changes to an entire healthcare system. These changes might not necessarily come from expected technological disruptions, but might come from the adaptation of the healthcare setting to certain methods and processes relying on AIHTs. This adaptation may initiate extensive and lasting transformations that are likely to affect all actors working in, using or financing the health system (Gerhards et al., 2020). The clinical use of some AIHTs may have the effect of transforming local health care administration practices by incorporating exogenous priors embedded within the technology. For instance, if a payer (public or private insurer) decides that a given AIHT recommendation become a precondition for reimbursement (i.e., making other care no longer reimbursable), this may have significant impacts on the way care is delivered, and will reduce patients’, clinicians’ and administrators’ autonomy in making shared and appropriate decisions when the human-recommended care is different than a new gold standard based on AI on data and priors (Vayena, Blasimme, and Cohen 2018). There is therefore a process of importing practices, potentially very different, which can strongly contrast with local habits and norms, requiring both adaptation and an impact assessment of these exogenous practices on the host environment.

AIHTs can have systemic real-world life-and-death consequences for patients (Miller, 2020), especially as AI will span across the life continuum from birth to death (Dzobo et al., 2020). AIHTs, unlike most drugs or medical devices, will also affect non-ill or non-frequent users of the health care system, be they due to AI increasing role in health surveillance, care optimization, prevention and public health, telemedicine (Love-Koh et al., 2018; Pesapane et al., 2018; Char and Burgart 2020). AI can help “democratizing health care” (i.e., in the sense of facilitating access) by extending care into patients’ homes (Reddy et al., 2020), places where more individualized and personalized care can be facilitated. While being increasingly present in patient care, AI will not address everything that has to do with the overall well-being of people. Some aspects less related to illness, such as spirituality and sociality, will most likely not be resolved and supported by AI systems (Dzobo et al., 2020). Therefore, a systematic response to using AI in health care may systemically neglect important aspects of care.

#### Harms and Tropism

Mistakes due to AIHTs used in clinical care and within the health care system have the potential to widely harm the patient population. Some AI systems, especially in primary care settings, can have impacts on the entire population of a hospital or clinic (such as an AI-powered patient triage). This makes some people say that it is all the more necessary that all algorithms should be submitted to extensive scrutiny, with an increased attention on validation in clinical settings before they can be deployed in medical practice (Dzobo et al., 2020). In addition, a key challenge in implementing AI is that, without a comprehensive understanding of health needs (especially those not covered by AI), there is a risk of fragmenting healthcare delivery by silo use of AI systems. Such silo use may lead to weakening health systems capacity and efficiency in addressing patients needs.

AIHTs can have tropism effects on the healthcare system that may shape and normalize certain practices and expectations that are not necessarily accepted, widespread, cost-effective or standard in new contexts. An example of this would be an AIHT trained on medico-administrative data in a context where physicians have often modified their billing to enter the highest paying codes for clinical procedures, causing the algorithm to infer that these codes represent the usual, standard, or common practice to be recommended (Alami et al., 2020). Thus, the algorithmic inference would be biased because the procedure billed maximizes the clinician’s remuneration, but potentially was not the one performed; this can lead to a cascade of non-cost effective recommendations. Such tropism effects may increase the risk of inappropriate treatment and care, and may result in importing AIHT-fueled standards and practices that are exogenous and non-idiosyncratic to local organizations and that may perpetuate latent biases in training data that are not present in certain health systems or contexts of care (Abràmoff et al., 2020; Alami et al., 2020; Miller, 2020). Therefore, the large-scale systematization of certain behaviors or inclinations may end up resulting in significant costs and harms for organizations and health systems as well for patients and HCPs (Alami et al., 2020; Hu et al., 2019). For example, higher sensitivities to clinical thresholds could lead to overdiagnosis or overprescription, while lower sensitivities could result in undiagnosed and untreated segments of the population; it is in the potential scope of the impacts that exceptionalism lies and must be carefully assessed in HTA (Alami et al., 2020; Topol 2020).

#### AI as a Transformation Lever for the Health Sector

According to Alami et al. (2020), instead of seeing AIHTs as a collection of distinct technologies, they should be regarded as a “health system transformation lever.” AI can serve as a strategic lever for improving health care and services access, quality and efficiency. Used in such way, AI could have significant society-wide impacts, including technological, clinical, organizational, professional, economic, legal, and ethical.

AI can become a key enabler of learning healthcare systems (LHS) to achieve their full potential (Babic et al., 2019), especially since AIHTs are themselves learning systems (Ho, 2020). AIHTs and LHS can complement each other as both strive when there are porous boundaries between research and development and with clinical and organizational practices. Using data from the health care system, AI can learn and recalibrate both its performance and behaviors, and over time inform and refine the practices of the health care system (Babic et al., 2019). AI can allow for ongoing assessment of accuracy and usage and continuous risk monitoring (Ho, 2020).

AI, as a lever, can also have a systemic impact of putting forward the response to needs for which there are ready-to-use technologies, causing to pay little attention to serious unmet needs (Alami et al., 2020). According to Grant et al. (2020), AI may represent the “next major technologic breakthrough” in health care delivery, offering endless possibilities for improving both patient care and yielding health care system-wide optimizations. However, this blurring of boundaries poses significant problems for adequate regulatory design and should not be taken lightly (Babic et al., 2019).

### Increased Expectations Towards Artificial Intelligence

Another key feature of AI exceptionalism is the increased expectations placed on AIHTs compared to other health technologies. According to Vollmer et al. (2020), AI systems often bear a misleading aura of obvious cutting-edge technology, which falsely limits the perceived need for careful validation and verification of their performance, clinical use, and general use once they begin to be used in routine clinical practice. The implications for HTA are three-pronged.

#### Belief that Since a Result Comes From an Artificial Intelligence It Is Better

A large part of the explanation for AI exceptionalism comes in particular from the belief that an AI-generated outcome is inherently better than a human one (Char and Burgart 2020). This phenomenon, known as the automation bias, describes the fact that slowly but surely, AI is establishing itself as an authority over current practices and error-prone healthcare professionals. Part of this is reflected in the fact that it is now recognized as a problem that a person who disagrees with a result or recommendation generated by an AI must justify their opposition with much more data than those used by the AI to achieve that result (Char and Burgart 2020). The technological imperative—i.e., the mere fact that a sophisticated technological intervention exists creates pressure to use it because it is perceived to be superior to conventional practices, despite the risks—reinforces this belief and AI in medicine is currently having its technological imperative moment (Carter et al., 2020).

#### Inevitability of Artificial Intelligence in Healthcare

These high expectations toward AIHTs form the basis of the inevitability of AI in health. To the point where AI is seen as inevitably the future of medicine (Dzobo et al., 2020). Self-learning and the ability to perform arduous and repetitive tasks explains the growing interest for a greater place of AI in standard medical care. There are high hopes and, according to many commentators, good reasons to think that in a near future, virtually all physicians will be assisted by AI applications to expedite certain tasks and, in the median term, due to continuous learning, AIHTs might outperform humans in a wide range of areas (Dzobo et al., 2020; Abràmoff et al.2020; Gerke et al., 2020). It is not only for clinical or therapeutic reasons that AI seems to be inexorable; there is also competition within the AI ecosystem. The growing importance of AI and its inevitability also stems from the competition between political decision-makers from different jurisdictions to widely deploy AI in order not to lag behind others (Gerhards et al., 2020). Considering all the interests at stake, the massive investments and accelerated development of AI, the question is no longer whether AI will be part of routine clinical care, but when (Reddy et al., 2020).

Although the technological imperative is strong and that AI in health is very attractive and seems inevitable, caution is called for. In this regard, AI chasm is a powerful concept to rebalance and help manage expectations of overly rapid deployment and ubiquity of AI in health care (McCradden et al., 2020). AI chasm refers to the phenomenon that while AIHTs are very promising, very few will actually be successful once implemented in clinical settings. HTA agencies have an important role to play here to contain this phenomenon and reduce its frequency and spread (McCradden et al., 2020). One of the roles of evaluation and regulation is precisely to finely consider the implications of these technologies to overcome this phenomenon. This requires not only an analysis of technical efficiency and performance, but also an oversight of empirical and ethical validation to ensure the rights and interests of patients. This requires the development of regulatory tools that are well adapted to AIHTs so that there are clear procedures and processes to properly evaluate and screen AIHTs (Alami, Lehoux, Auclair, Guise, et al., 2020; Abràmoff et al., 2020). This is necessary to avoid ethical drifts and unacceptable (economic, health, social) costs that may be caused by technologies that are not adapted to the needs and specificities of a clinical or organizational context, or by milieus feeling pressure to deploy a technology and adapt its practices in a way that ultimately does not benefit patient care or sound health care management (Michie et al., 2017; Abràmoff et al.2020).

#### Navigating the Hype

AI is currently in an era of promises rather than of fulfillment of what is expected from it (Michie et al., 2017). This new science has yet to move beyond average outcomes on individuals to actual personalized benefits based on their situation, characteristics, and desired outcomes. It is important to remain critical and vigilant with respect to the rush to adopt these new technologies, possibly more so than politicians are at present (Cowie 2017). Especially when thought leaders’ perspectives echo public wonderment and aspirations that AI transforms human life (Miller, 2020). With their development and implementation being largely driven by a highly speculative market and by proprietary interests, AIHTs are largely embedded into biocapital (Carter et al., 2020). That is to say, a vision of medical innovation that is based not on the actual creation of value, but on selling a certain vision of the future. It is through the sale of imaginary evoking unparalleled performance and disproportionate benefits to encourage all AI players to engage in the massive implementation of AI despite its uncertainties and shortcomings (Carter et al., 2020). That being said, in a study reported by Vayena et al. (2018), half of the surveyed American healthcare decision-makers expect that AIHTs will both improve medicine and fail meeting hyped expectations. Miller (2020) sums up the present phenomenon as follows: “No matter how sanguine the gurus touting game-changing AI technologies are, and no matter how much caregivers and patients hope that their benefits to medical practice and outcomes are not hype, all parties must remain vigilant.” AIHTs are in their phase of promises and hype, which is creating inconsequent expectations (Reardon 2019).

The consequences of these unreasonable expectations can be very significant. For instance, patients’ unsound expectations regarding the clinical outcomes of AIHTs can negatively affect their care experience (Alami et al., 2020). Certain areas of care, such as breast cancer, are particularly fertile ground to AI companies’ hype and promises (Carter et al., 2020), because it resonates with patient unfulfilled demands. The counterweight to these expectations is not yet in place as, despite all the hype, the science of AI is still young and possibly not yet mature, including gaps in clinical validation and perhaps imprecise health recommendations (Dzobo et al., 2020). HTA agencies and regulators have an important role to play, particularly in developing a regulatory infrastructure that is as exceptional as technology can be for health and as powerful as the “unfounded hype” can be, to use Mazurowski (2019) expression.

### New Ethical Challenges

There seems to be broad agreement that AIHTs present new ethical challenges (Vollmer et al., 2020). According to Michie et al. (2017), AIHTs presents “new challenges and new versions of old challenges” which require new evaluative methods and legislative motivation to address health data and AIHT-specific ethical and regulatory issues.

#### Health Care Delivery

Reddy et al. (2020) identified three key AIHT-stemmed ethical challenges in care delivery: AI-fostered potential bias, patient privacy protection and trust of clinicians and the general public towards machine-led medicine. AI is also prone to generating new health inequalities; perhaps more potent than its ability to reduce existing ones (Fenech et al., 2020). An important caveat in terms of health care equity comes from the fact that those who compare very well with historic patient data will be the one benefiting the most from AIHTs. A cautious attitude is therefore called for with regards to patient and disease heterogeneity, taking into account that patterns detected by AI are largely deduced from smaller historical data sets (Dzobo et al., 2020). In addition to the current disparities, digital literacy and access to technologies are adding up, so that if nothing is done, large segments of the population may be excluded from enjoying the benefits of AIHTs, resulting in significant issues of justice (Fenech et al., 2020).

#### Existential Questions

According to Fenech et al., 2020, AI is unlike any other health technology, due to its capability of being a general-purpose technology forcing to question the very essence of humans. This technology is particularly sensitive for the healthcare sector as it raises new existential questions that regulators and public decision-makers must now face. One of such key existential challenge for HTA, according to Haverinen et al. (2019), is that AI is becoming a new decision-maker. This adds an actor with a decision-making role on the fate of patients and the health care system in addition to the role of HCPs and increases the complexity of performing comprehensive HTA. For Ho (2020), a distinctive ethical concern that stems from AIHT is the technology’s unparalleled autonomy, which intensifies ethical and regulatory challenges, especially in terms of patient safety. While this obviously raises questions about liability (who is at fault for harm, and who is responsible for explaining it and being accountable to patients), it also requires thorough thinking about appropriate ways to ensure that care is humane and respects the dignity of persons (Pesapane et al., 2018; Vayena et al., 2018; Fenech et al., 2020).

#### Challenging Medical Ethics’ Ethos

Exceptionalism also stems from the fact that the field of medicine is structured around the transparency and explainability of clinical decisions, which poses serious problems for the acceptance, regulation and implementation of (too often inexplicable) AI in the health care system (Reddy et al., 2020). As Miller (2020) points out that technology insertion is never neutral, both the success of AI in health care and the integrity and reputation of health care professionals depend on the alignment between the ethos of medical ethics and the ability of AIHTs (notwithstanding its benefits and performance) to respond to the challenges that its very characteristics pose to the health care system (Reddy et al., 2020). It is therefore widely acknowledged that AI will have considerable benefits on health care (optimized process, increased quality, reduced cost, and expanded access) that will come at the price of raising ethical issues specific to the technology (Abràmoff et al., 2020). This moral cost and related ethical considerations partly explain that the field of AI ethics has recently “exploded” as academics, organizations and other stakeholders have been rushing to examine the ethical dimensions of AI development and implementation (Fiske et al., 2020). However, some are skeptical, such as Fenech et al. (2020) who was warning that data ethics is fashionable. While Bærøe et al. (2020) go as far as arguing that “exceptional technologies require exceptional ethics” and that “an intentional search for exceptionalism is required for an ethical framework tasked with assessing this new technology”.

### New Evaluative Constraints

According to Dzobo et al. (2020), by being very distinct from more traditional technologies, AI must be regulated differently. Zawati and Lang (2020) argue that the uncertainty regarding AI decisional processes and outcomes make AIHTs particularly challenging to regulate. Regulators, policy-makers and HTA agencies are faced with unprecedented complexity for evaluating and approving AI (Alami et al., 2020). AIHTs raise new evaluative constrains, be they technological, clinical, organizational, that affect how ethical, legal and social dimensions may be tackled (Gerke et al., 2020). Evaluation constraints are related to data, real-world uses and the embryonic nature of the regulatory infrastructure and processes.

#### Data-Generated Issues

AI uses larger than ever volumes of data generated by individuals, governments, and companies, and according to Fenech et al. (2020), the greater complexity of health data raise new questions about the governance of data use and storage, especially as AI technologies are only effective and relevant with up-to-date, labelled, and cleaned big data. In addition to data, the hardware infrastructure will need to be updated over time, requiring major financial investments to maintain the use of AI in the healthcare system (Dzobo et al., 2020). However, too few technical studies are helping to appreciate and help managing AIHTs’ complexity. In most studies, contextual, clinical and organizational considerations, implementation and uses of the technology are neglected, which complicates regulators’ assessment as they are mostly informed about the significance of AI applications’ technical performance (Alami et al., 2020). Caution should therefore be exercised, particularly since the complex ethical and regulatory issues involved deserve careful consideration before deploying these technologies in routine clinical care (Prabhu 2019).

#### Real-World Usages and Evidential Issues

AIHTs raise new regulatory challenges in part due to the greater variation in their performance between the test environment and the real-word context than those of drugs and medical devices. According to Gerke et al. (2020), AIHTs have potentially more risks and less certainty associated with their use in real-world contexts, which is central to regulatory concerns. However, most AIHTs have not been objectively validated in and for real-world usages (Alami et al., 2020). In that sense, one important caveat is that the adoption and impact of AIHTs are unlikely to be uniform or to improve performance in all health care contexts (Gerke et al., 2020). This is attributable concurrently to the technology itself (and its distinctive features that renders it disruptive), to the contexts of implementation (the systemic impact of the technology across the health care system, and clash with local practices) and to the human biases associated with the use of these technologies (inability to reason with AI-provided probabilities, small samples and noise induced extrapolation and false patterns identification, and undue risk aversion) (Gerke et al., 2020). For regulatory authorities, these represent great challenges for deciding whether marketing authorization is justified. But it is also puzzling for hospital, clinic and health care system purchasers to determine whether an AIHT will actually add value and increase performance of care and service delivery. There is a lot to be studied and understood on the broad systemic policy implications of AIHTs in real-world context of care and services (Alami, Lehoux, Auclair, de Guise, et al., 2020).

#### Undeveloped Regulatory Infrastructure and Processes

AIHTs’ exceptional characteristics have significant regulatory implications as regulation is emerging, but at a far slower pace than technological changes, which are virtually infinite (Char and Burgart 2020). Regulative complexity is furthered by the fact that existing standards (e.g., those of the Food and Drug Administration, European Medicines Agency, Health Canada) do not translate well for self-evolving technologies (Dzobo et al., 2020; Shaffer 2020; Topol 2020). This definitional deficit complicates the regulation of this technology and the implementation of appropriate policy infrastructure (Pesapane et al., 2018). Recent approvals of algorithms highlighted some limitations of existing regulatory standards and processes (Haverinen et al., 2019; Parikh et al., 2019). These considerations are threefold and concern the levels of requirements, the speed of AIHT developments and the equilibrium posture that regulators must adopt.

A challenge for existing regulatory regimes lies in the extensive information requirements on both the nature and effects of health technology, as well as clinical data on efficacy and patient safety, and population and societal impacts. However, regulators have yet to develop an infrastructure and processes that are appropriate and optimal for AI, and this requires more knowledge about how algorithms work (Dzobo et al., 2020). This complicates the problem because AI is a less transparent and explainable technology than drugs or medical devices can be (Abràmoff et al., 2020; Reddy et al., 2020). Privacy concerns are also important and there is yet no public agreement regarding data collection and sharing for commercial purposes, nor regarding for-profit data ownership (Michie et al., 2017). This calls for finding collective responses to these considerations, which must accompany the work of structuring HTA practices and infrastructures by regulators (Fenech et al., 2020).

Another dimension putting pressure on regulation is the speed of development. For regulating a fast-changing and unpredictably sector such as AI, time is of the essence to ensure that regulatory standards and practices are relevant (Pesapane et al., 2018). Currently, regulation has to constantly catch up with the private sector which leads to important gaps in terms of ethical examination of AIHTs (Shaffer 2020). Since, most developments are done by the private sector and HTA processes are not yet well designed and optimized, there is a problem of scrutiny (Abràmoff et al., 2020). So to keep up, regulation must be as fast as technological developments in AI, therefore it requires to conduct assessment and oversight at an unprecedented pace (Haverinen et al., 2019). However, this need to proceed quickly, in particular to match the private sector’s pace, must be put into perspective with the very acceptability of a significant presence of commercial interests in the big data and AIHTs sector.

Achieving the right balance is delicate for HTA agencies between accelerating the development of HTA policies and procedures and not falling prey to the sirens of AIHT’s hype (Cowie, 2017; Miller, 2020). Regulators want to see the health care system reap AI’s benefits quickly, but if their assessment is too hasty and the implementation of the first generations of AIHTs encounters difficulties or, worse, generates adverse effects, social and professional acceptability may be shattered and further delay the deployment of AI in healthcare. According to Reddy et al. (2020), it could need a single serious adverse incident caused by an AIHT to undermine the public’s and HCP communities’ confidence. Considering that AI’s acceptance is still fragile, and that AI is expected to have an expanded presence in all aspects of the health care system, HTA agencies will have to be extra careful in considering the ethical and regulatory implications of IA. If not well managed, these considerations will become major barriers that will play against the deployment of AI in healthcare (Pilotto et al., 2018; Vayena et al., 2018; Bærøe et al., 2020).

## Discussion

The current body of literature appears to portray AI health technologies as being exceptional to HTA. This exceptionalism is expressed along five dimensions. Firstly, the very nature of the technology seems to be the primary cause of the difficulty in fitting AIHTs into current HTA processes. Thus, the still ambiguous definition and the consequences of its changing and evolving nature pose new challenges for its assessment. Secondly, the scope of its impact far surpasses those of current health technologies. AIHTs will have impacts that extend significantly beyond the targeted patients and professionals. It is therefore in the interest of HTA agencies to consider the disruptive effects on individuals as well as on the entire health care system. Hence, the importance for HTA to consider the potential harms, the systematization of biases and to anticipate the clashes between current practices that are working well and the framing effect that will come with the deployment of AIHTs. But also, AI can act as a transformational lever that, beyond the risks of AI in healthcare, appears to be capable of redressing and reorienting the healthcare system to better respond to the full range of healthcare needs, to create synergies so that the of learning healthcare systems are operational and to take this opportunity to adjust the regulatory design. Third, the advent of AI in healthcare comes with a lot of high expectations. The quality of outcomes generated by AIHTs is expected to be higher than that of current human-driven processes. This positive perception of the added value of AIHTs in the healthcare system makes AI in healthcare appear inevitable. However, while the technology is currently casted as exceptional and highly promising, some caution should be kept towards the current hype, which should prompt regulators to be prudent towards unreasonable expectations. Fourth, AIHTs are challenging HTA from an ethical perspective as AI has a strong potential to generate greater inequity whether arising from algorithmic decisions or in access to AIHTs. The fact that AIHTs are becoming new decision-makers, due to their autonomy, raises important issues of patient safety as well as liability. *In fine*, medical ethics’ ethos is even shattered since, with AI, ethical dilemmas seem to be amplified, calling potentially for ethical standards revamping that would be as exceptional as the technology. Finally, AI technologies in health are increasing regulatory complexity and are pressuring current HTA structure and processes. The new evaluation constraints relate to data, real-world uses and the rather embryonic nature of AI-ready regulatory infrastructure and processes. Therefore, be they the extensive information requirements on both AIHTs’ features and effects for regulatory review, the speed of AIHTs’ developments, and the need to regulate quickly, but in a way that benefits the entire population.

A key point emerging from the views of the authors reviewed is that *exceptionalising* views of AIHTs, in the context of HTA, appear to come as much from the technology itself as they do from the broader social, cultural, and political contexts surrounding AI in the health sector. In other words, AIHTs are exceptional because of their technological characteristics *and* potential impacts on society at large. This is quite consistent with HTA, which seeks to assess the diversity of impacts of a technology. It is therefore quite reasonable that a technology with multi-dimensional impacts on society severely affects a process that is based on these same dimensions. The key takeaway may be that, to adapt and remain relevant, HTA must continue to focus on and strengthen these evaluative processes, which must be capable of a comprehensive assessment of the technical, social, cultural, ethical and health dimensions.

Interestingly, no author in the reviewed sample clearly promoted the idea that AI is an unexceptional technology for HTA. Many reasons could explain this phenomenon. First, the hype is still very strong when it comes to AIHT (Mazurowski 2019; Carter et al., 2020). Thus, it is possible that discussions about AI (un)exceptionalism are not yet ripe to mark the literature. This can be seen in the literature reviewed, which, without focusing on the limits of exceptionalism, currently has its strongest criticisms on AI hype. This leads us to think that hype and exceptionalism may be linked: hype feeds on exceptionalism while the latter needs hype to surface and to strike a chord within the literature and the rhetoric about AI in health. Second, trivially, this may be because there is less incentive to write (and publish) on the advent of a new technology in health by stating that nothing is new under the Sun (Caulfield 2018). Third, AIHTs may be so recent in the HTA pipeline that HTA as not yet addressed all the dimensions of AIHT.

Even if AI’s exceptionalism appears significant in the current body of literature, there is some caveats to promoting AI exceptionalism in HTA. First, two authors noted some limitations to AI exceptionalism. Michie et al., (2017) pointed out that AIHTs are not only raising new challenges, they also bear issues that are common to existing health technologies. This is echoed by Char et al. (2020) who acknowledge the phenomenon, but puts a limit to the enthusiasm for AI exceptionalism in health when it comes to AIHTs sporting some features similar to standard health technologies. Second, currently, the literature discussing AI exceptionalism is still piecemeal, and it would be relevant for future research to address the issue by looking more holistically at the full range of issues posed by AI (i.e., outside the sole realm of HTA). There is still some space to apprehend and analyze the exceptionality of AIHTs’ in HTA and the implications this has for both the evolution of HTA and the development and use of AIHTs. The literature is still quite young, and this can be observed from the fact that some highly discussed considerations in the broader AI ethics and AI in medicine literatures have not been discussed in the body of literature at review. For example, the more structural implications related to data-generated issues—privacy, data stewardship and intellectual property (Bartoletti 2019; Cohen and Mello 2019)—or to issues pertaining to informed consent, patient autonomy and human rights (Sparrow and Hatherley 2019; Cohen 2019; Racine et al., 2019; Ahmed et al., 2020), or to human-machine comparison in medical decision making and diagnosis (London 2019) have not been explored in the studied subset. A comprehensive exploration of the themes generally associated with health technologies will provide a better understanding of and clarity on whether AIHTs are exceptional or not. Third, exceptionalism in the context of health innovation is not a new topic. New health interventions or discoveries often generate a lot of hype, and the sector is hungry for predictions about the next revolution in healthcare (Emanuel and Wachter 2019). Twenty years ago, the health sector was living the “genomic revolution” and was assessing the exceptional implications of genetics in healthcare (Suter 2001). As AIHT’s literature mature, it may continue to be centered on its exceptionality; but it is also plausible to consider that, as with the genetic revolution in the early 2000s and the nanotechnologies in the 2010s, AI exceptionalism will pass what Murray (2019) calls its “sell-by date”. Thus, AI exceptionalism may end up following a rather similar pattern where the hype will slowly wear off as the health sector will become more accustomed to the technology; better understand its actual strengths, limitations, and capabilities.

Therefore, possibly it is a matter of time before a coherent body of literature addresses the limits of an *exceptionalist* view of AI in health and HTA. At the same time, if the AI revolution really takes off, exceptionalism will no longer be an important consideration. Indeed, as regulatory systems, the healthcare system and human agents (clinicians, patients, regulators, managers, etc.) adapt, exceptionalism will probably pass and habituation will make AI in health the new normal, as after any major disruption that lasts over time. However, it may be of interest to the AI, HTA and health regulation communities and scholars to remain vigilant about AIHTs’ exceptionalism (by means of the manifestation of its five dimensions) in health and HTA.

Another avenue that the literature could explore is whether the exceptionality arises from the technology (i.e., AI) or the sector of application (i.e., health)? In other words, is it AI that is exceptional in health or rather health that is a sector of exception for AI? Healthcare is possibly the most regulated sector that AI has come across so far. Health’s exceptionality may explain the significant regulation in the healthcare sector (i.e., attention given to this sector in terms of regulation, ethics, law, and society) (Daniels 2001; Bird and Lynch 2019), while no other sector has an assessment process that has the breadth and systematism of HTA. Therefore, it would be interesting to reverse the question at the very basis of this review and consider how, for the AI ecosystem, health is *per se* exceptional and calls for additional and distinct norms, practices and contingencies that need to be considered to develop and implement an AIHT. Thereby, in addition to offering insights and guidance to communities strongly engaged in HTA, our results can also help the AI research and development sector better understand the unique evaluative considerations that exist in the health sector. The five dimensions raised by our paper can help guide those developing AIHTs to better understand and respond to the specific expectations and priors that underlie the use, administration and acceptability of health technologies. This can potentially help better align AIHT developers’ desire to create value with HTA agencies’ value appreciation and thus facilitate the congruence between technology development and healthcare priorities (Chalkidou 2021).

More broadly, the literature review raises key institutional questions, in terms of the exceptional issues posed by AI, such as which body is best placed to incorporate the new and added concerns that AIHTs raise? Is it the (supra)national regulators (e.g., EMA, FDA, Health Canada and the like which are mostly responsible for evaluating safety, efficacy, and quality concerns) or the HTA bodies (who are more concerned with appropriate use, implementation, coverage and reimbursement)? Can certain issues (e.g., the ethical and social ones) be better addressed by one body versus another? A possible limitation of the literature review is that overall the authors do not generally make a clear distinction between the regulatory processes (those of the FDA, EMA, and Health Canada that aim to allow the marketing of AIHTs) and the HTA (which focus on assessing implementation, optimal use, and whether or not to recommend reimbursement by third-party payers), so it was not possible to specify the unique considerations that arise specifically for either or both. One thing is for certain, the exceptional challenges of AIHT further raise the importance, for regulators and health technology assessors, to consider the impacts of AI uses in healthcare in a holistic way. This points to pivoting current rather linear regulatory and HTA process towards a “lifecycle” approach, which would allow for a better consideration of the five exceptional dimensions of AIHT. This may sound demanding, but AIHTs already represent additional evaluative burdens, especially when it comes to long-term real-world usages (e.g., when AIHT are used on new populations or for new purposes that differ from the data on which it was trained, or simply behave differently from what was expected at the time of the regulatory or HTA assessment) and the difficulties of withdrawing AIHT from the market. This calls for more cooperation between regulators and HTA agencies, but also hint towards a global health technology governance reform to allow increased scrutiny capability, and also to help AIHT regulatory and HTA assessment adjust overtime (i.e., by using a lifecycle approach) based on the evolving (clinical, economic, social, ethical) evidence.

In any case, there is a strong argument for taking into consideration the exceptional aspects of AIHTs, especially as their impacts on the healthcare system will be far greater than that of drugs and medical devices (Vayena et al., 2018; Babic et al., 2019). As AI applications begin to be much more readily introduced into the health care sector, there is a window of opportunity for HTA agencies and scholars to consider the broad spectrum of impacts that AIHTs may generate (Bostrom and Yudkowsky 2011; Helbing 2015; Burton et al., 2017; Herschel and Miori 2017; Knoppers and Mark Thorogood 2017). AI implementation by governments and health organizations carries risks of generating new and amplifying existing, challenges due to a shift from the current mostly human-driven systems to new algorithm-driven systems (Vayena et al., 2018; Zafar and Villeneuve 2018; Reddy et al., 2020). Hence the need to address the distinct (without the need for them to be exceptional) characteristics of AIHTs to inform HTA developments in a way to ensure that only clinically, economically, socially acceptable AIHTs are deployed in the health care system.

## Conclusion

Therefore, is it possible to assert that there is such thing as an AI exceptionalism in HTA? It may be too early to be decisive on this issue, although the literature reviewed seems to point in this direction. Our review of the literature has allowed to identify five dimensions through which AIHTs are exceptional, from an HTA standpoint: nature, scope, increased expectations, new ethical challenges and new evaluative constrains. Most importantly, what underlies the promises of AI, the hype, and the exceptionalism is that we are mostly in an era of speculation; while some applications have begun to work their way into the healthcare system, the much-anticipated revolution is still a ways off. It is the test of time that will determine the veracity and breadth of the exceptionalist perspective. But whether or not exceptionalism proves to be valid, HTA must certainly adapt to the massive arrival of AI in health. This must be done by considering and responding to the five dimensions of exceptionalism and their many implications that can undermine the appropriateness, efficiency, and relevancy of current and future HTA infrastructure and processes. Our results should help inform where HTA stakeholders need to pay special attention and adapt their policy architecture and processes so that they become agile to adopt a regulatory posture capable of appreciating the distinct characteristics and impacts that AIHTs pose in the health sector.

## Data Availability

The raw data supporting the conclusions of this article will be made available by the authors, without undue reservation.
